# Supplementary material for: The Synthesis, Characterization and Anti-Tumor Activity of a Cu-MOF Based on Flavone-6,2′-dicarboxylic Acid
Source: Molecules. 2022 Dec 23;28(1):129. doi: 10.3390/molecules28010129 (PMC9822075; doi:10.3390/molecules28010129)
Supplement: Supplementary file 1 [file molecules-28-00129-s001.zip › molecules-2069559-supplementary.pdf]

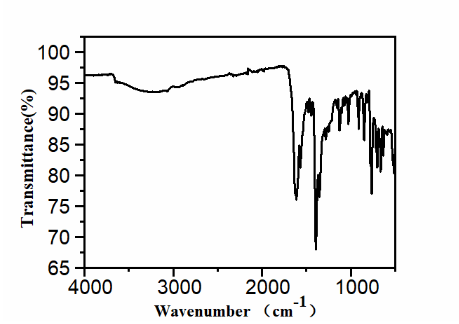

Figure S1. IR spectrum of LDU-1

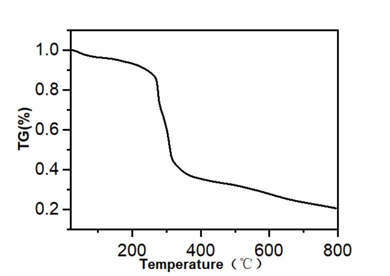

Figure S2. TG curve of LDU-1

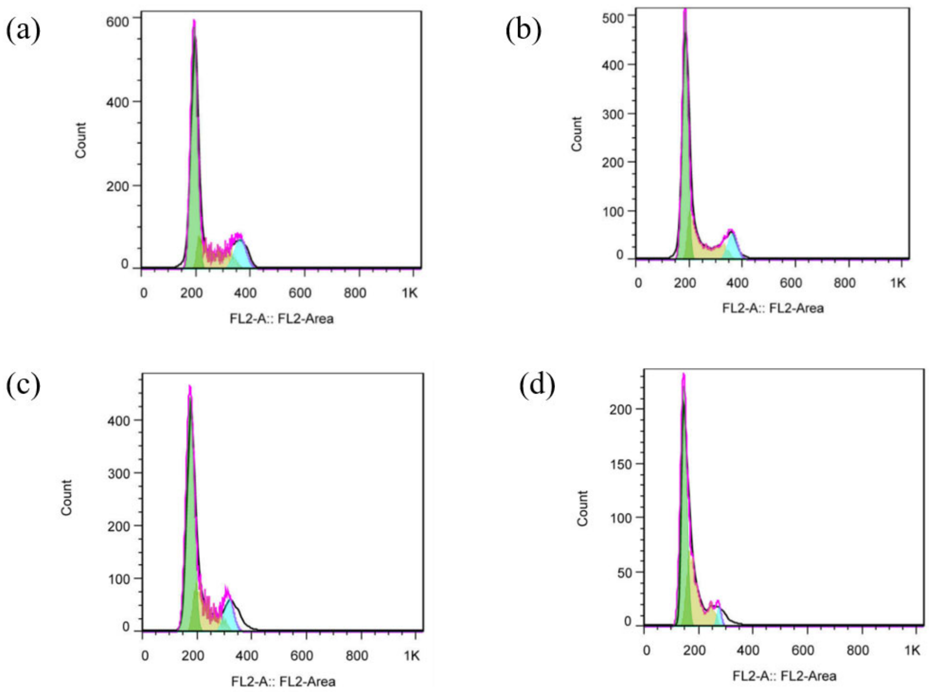

Figure S3. Cell cycle phase distribution of the A549 cells as measured by flow cytometry after treatment with PBS(**a**), cisplatin(**b**), H_2L(**c**) and LDU-1(**d**).

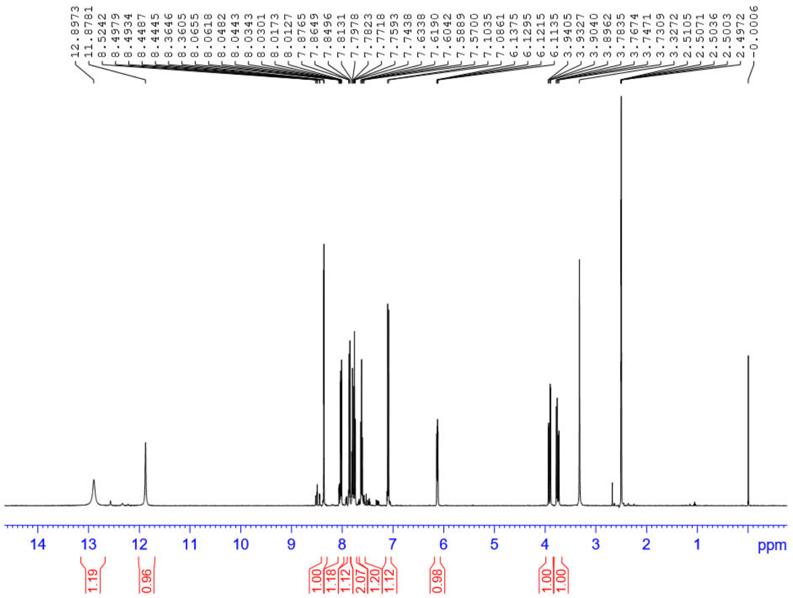

Figure S4. ^{1}H NMR of 2-hydroxychalcone-2,5'-carboxylic acid(**2**)

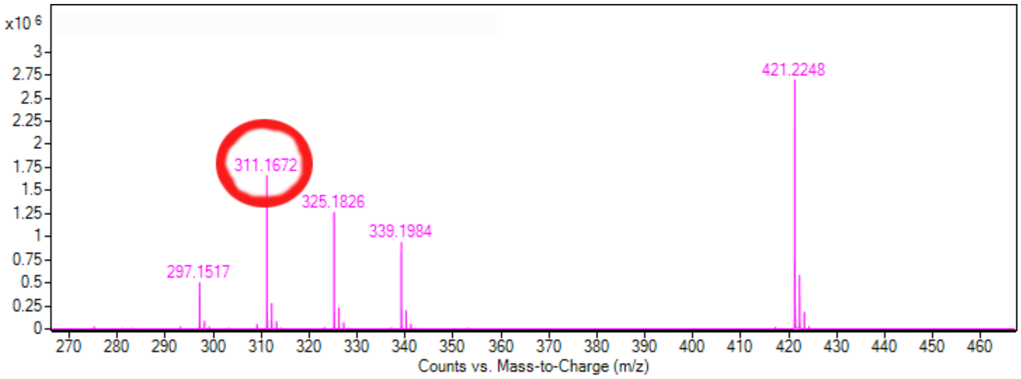

Figure S5. Mass spectrum of H_2L

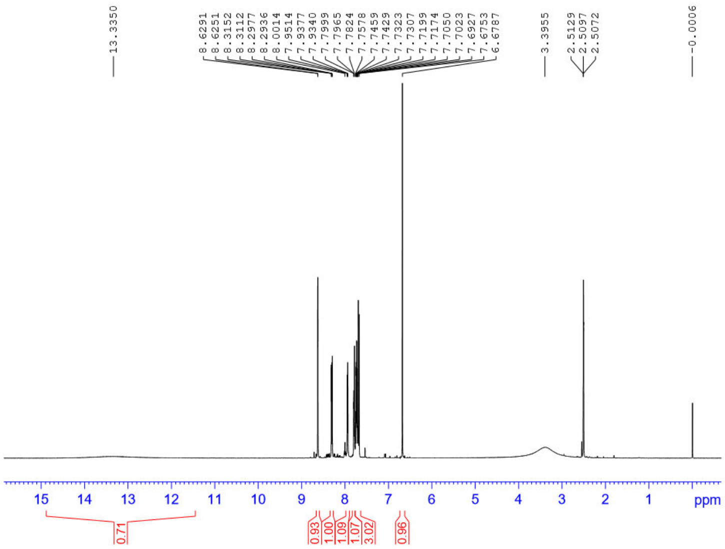

Figure S6. ^{1}H NMR of flavone-6,2'-dicarboxylic acid(H_2L)

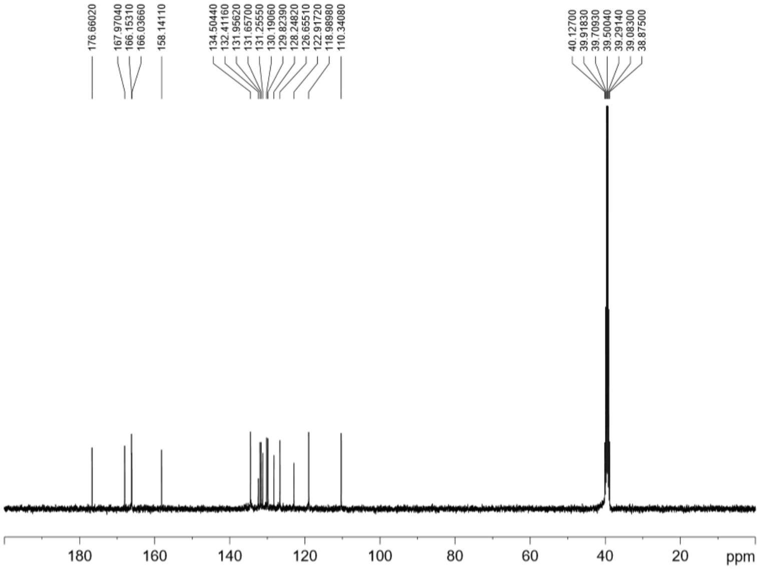

Figure S7. ^{13}C NMR of flavone-6,2'-dicarboxylic acid(H_2L)
